# Case report: Reconstruction of the long-gap unilateral absence of right pulmonary artery with contralateral pulmonary artery flap and autologous pericardial graft

**DOI:** 10.3389/fcvm.2023.1071111

**Published:** 2023-03-08

**Authors:** Tiange Li, Yunfei Ling, Ziqing Xiong, Qi An

**Affiliations:** Department of Cardiovascular Surgery, West China Hospital of Sichuan University, Chengdu, China

**Keywords:** unilateral absence of pulmonary artery (UAPA), congenital heart defects, cardiovascular surgery, reconstruction of pulmonary artery, surgical techinque

## Abstract

Unilateral absence of pulmonary artery (UAPA) is a rare type of congenital abnormality that may coexist with other congenital abnormalities or present as an isolated lesion, the latter form can be asymptomatic. Surgical procedure is usually carried out when UAPA was diagnosed with significant symptoms, and the aim of surgery is to restore the pulmonary flow distribution. The right-side UAPA is a considerable challenge for surgeons to process surgery, however, technical description of this type of UAPA are limited. Here we described a rare case of a two-month girl with absence of right pulmonary artery, we presented a technique that reconstructs this long-gap UAPA with contralateral pulmonary artery flap and autologous pericardial graft.

## Introduction

Unilateral absence of the intrapericardial pulmonary artery (UAPA) is a rare congenital anomaly arising from proximal pulmonary arterial obstruction with the closure of the ductus arteriosus. Due to difficult diagnosis and mild early symptoms, the exact prevalence remains unknown, though the reported prevalence of isolated UAPA without associated cardiac anomalies ranged from 1 in 200,000 to 1 in 300,000 adults (1, 2). The primary treatment goal is to restore the pulmonary flow distribution as close to normal as possible, which can be achieved through surgical continuity. Previous studies have provided various surgical designs to correct UAPA, such as direct anastomosis of the affected pulmonary artery (PA) to the main pulmonary artery (MPA), reconstructing PA with autologous pericardial roll, pulmonary artery or prosthetic materials, creating a systemic-to-PA shunt, repairing absent PA with a contralateral PA autograft segment interposition, using an in-situ pedicle of transverse sinus pericardium as a base for a conduit to reconstruct PA and so on (3–7). The right-sided UAPA, however, poses a significantly greater surgical challenge compared to the left-sided UAPA due to the relatively long distance between the right pulmonary artery (RPA) and the MPA. Currently, technical descriptions of absent RPA reconstruction are limited. Here, we describe our technique of modified PA flap angioplasty used to reconstruct the right-sided UAPA with long-gap discontinuity that permits tension-free anastomoses while also potentially mitigating the risk of long-term stenoses.

## Case presentation

**Figure 1:**
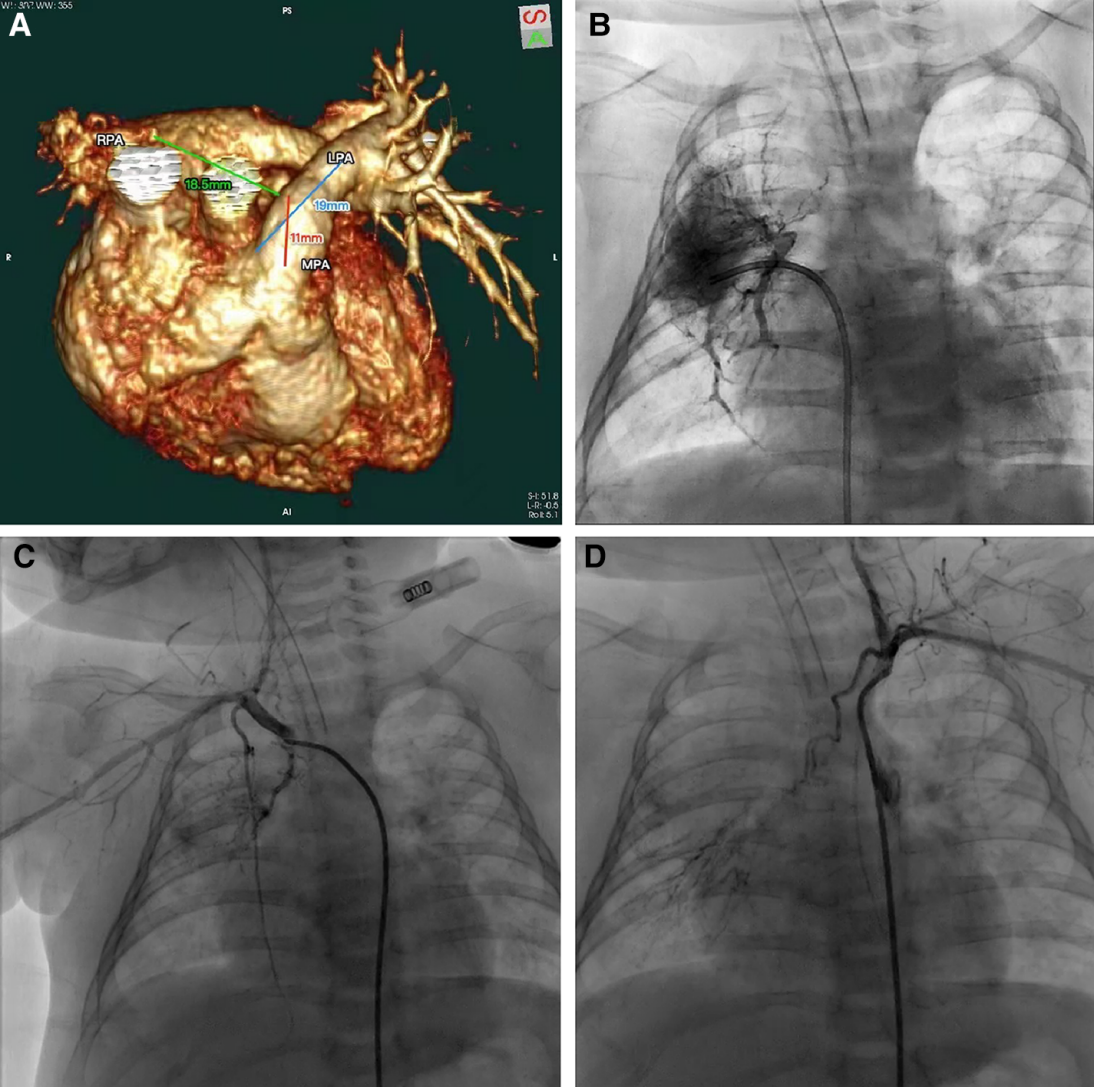
(**A**) CTA and three-dimensional reconstruction revealed the absence of intrapericardial right pulmonary artery. (**B–D**) Cardiac catheterization revealed blood supply of upper right pulmonary artery comes from right subclavian artery and inferior right pulmonary artery was supplied by left subclavian artery.

Our patient was a 2-month-old girl first presented with convulsion due to pneumonia at 4 days old. No obvious cardiac abnormality was discovered upon initial physical examination. Echocardiography at the time suggested the absence of RPA, a patent ductus arteriosus, a patent foramen ovale and pulmonary hypertension. She was then transferred to our institution for further treatments. Preoperative computer tomography angiography (CTA) and three-dimensional reconstruction revealed the absence of intrapericardial RPA, of which the diameter of distal RPA at hilus is normal. The diameters of MPA and left pulmonary artery (LPA) was 8.3 mm and 5.1 mm respectively (with z-scores of −1.25 and −0.83 respectively). The gap between the RPA at the hilum and MPA was estimated to be 18.5 mm (Figure 1A). We further performed cardiac catheterization for diagnostic purposes, which further revealed that blood supply of upper RPA comes from right subclavian artery, while inferior RPA was supplied by left subclavian artery through long collateral vessels (Figures 1B,D). Systolic/diastolic pressure of the ascending aorta (AAo), superior vena cava (SVC), MPA and RPA were 64/29 mmHg, 20/7 mmHg, 51/25 mmHg and 20/10 mmHg respectively.

**Figure 2:**
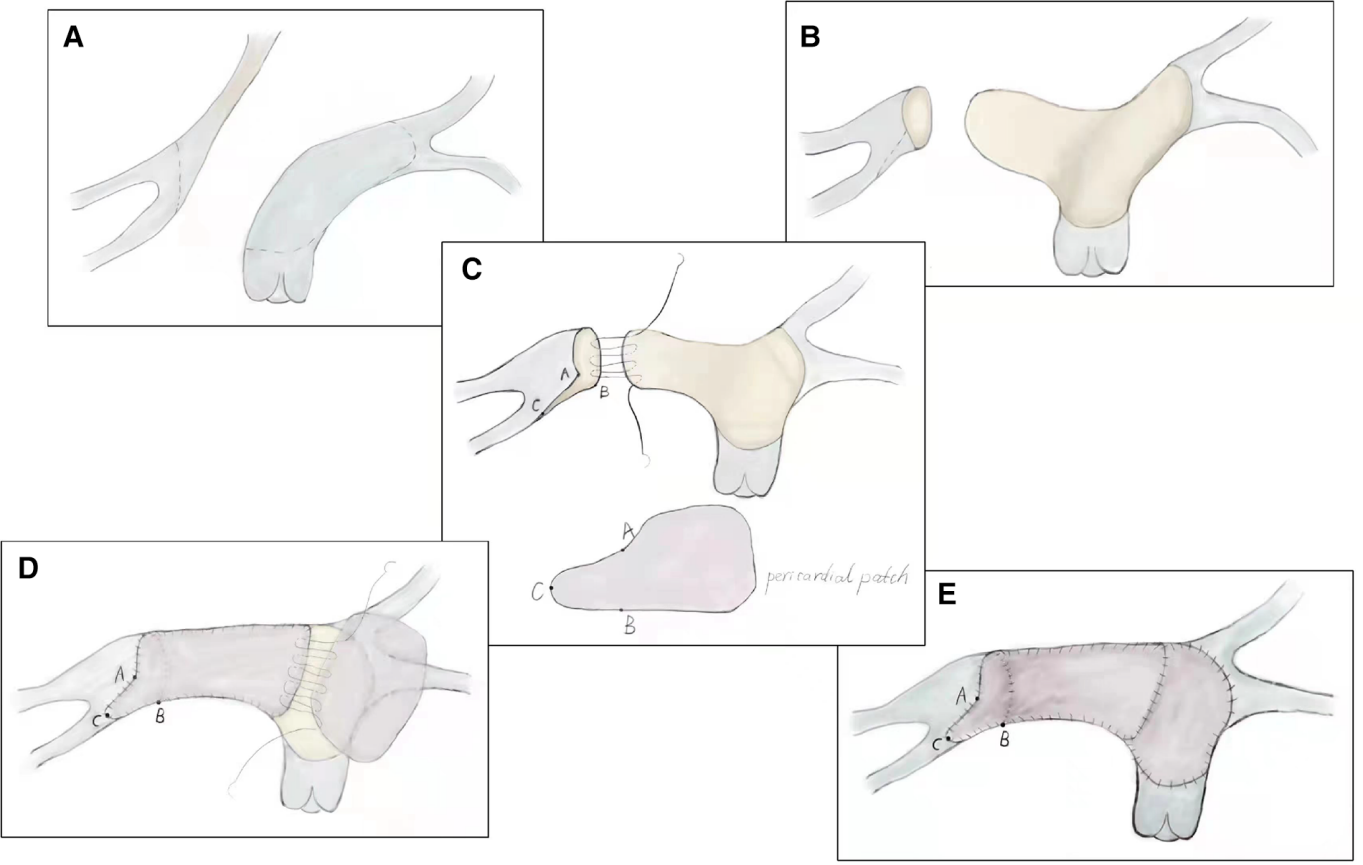
Technique of modified pulmonary artery flap angioplasty. (**A**) Extension flap was created using the anterior wall of main pulmonary artery and left pulmonary artery. (**B**) Distal incision for the extension flap terminated proximal to the left pulmonary artery hilum. (**C**) Incision was made along the inferior aspect of the right lower lobar branch to enlarge the right pulmonary artery hilum. (**D**) Inferior, anterior, and superior aspects of the neo-right pulmonary artery were reconstructed using a glutaraldehyde-treated autologous pericardial patch. (**E**) Anterior wall of main pulmonary artery and left pulmonary artery was reconstructed with a second treated autologous pericardial patch.

**Figure 3:**
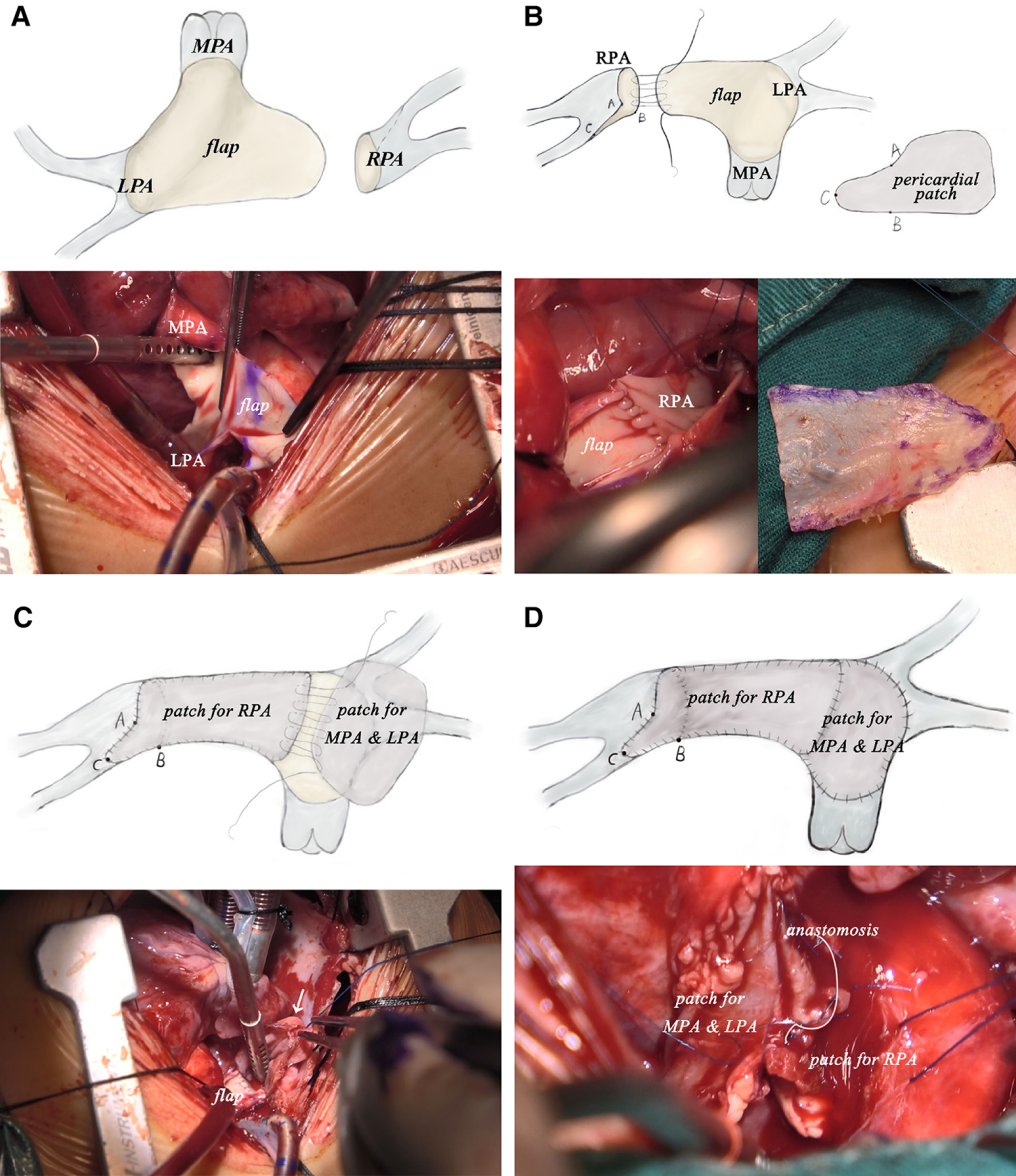
Intraoperative pictures of modified pulmonary artery flap angioplasty corresponding to procedure diagram. (LPA, left pulmonary artery; MPA, main pulmonary artery; RPA, right pulmonary artery.

**Figure 4:**
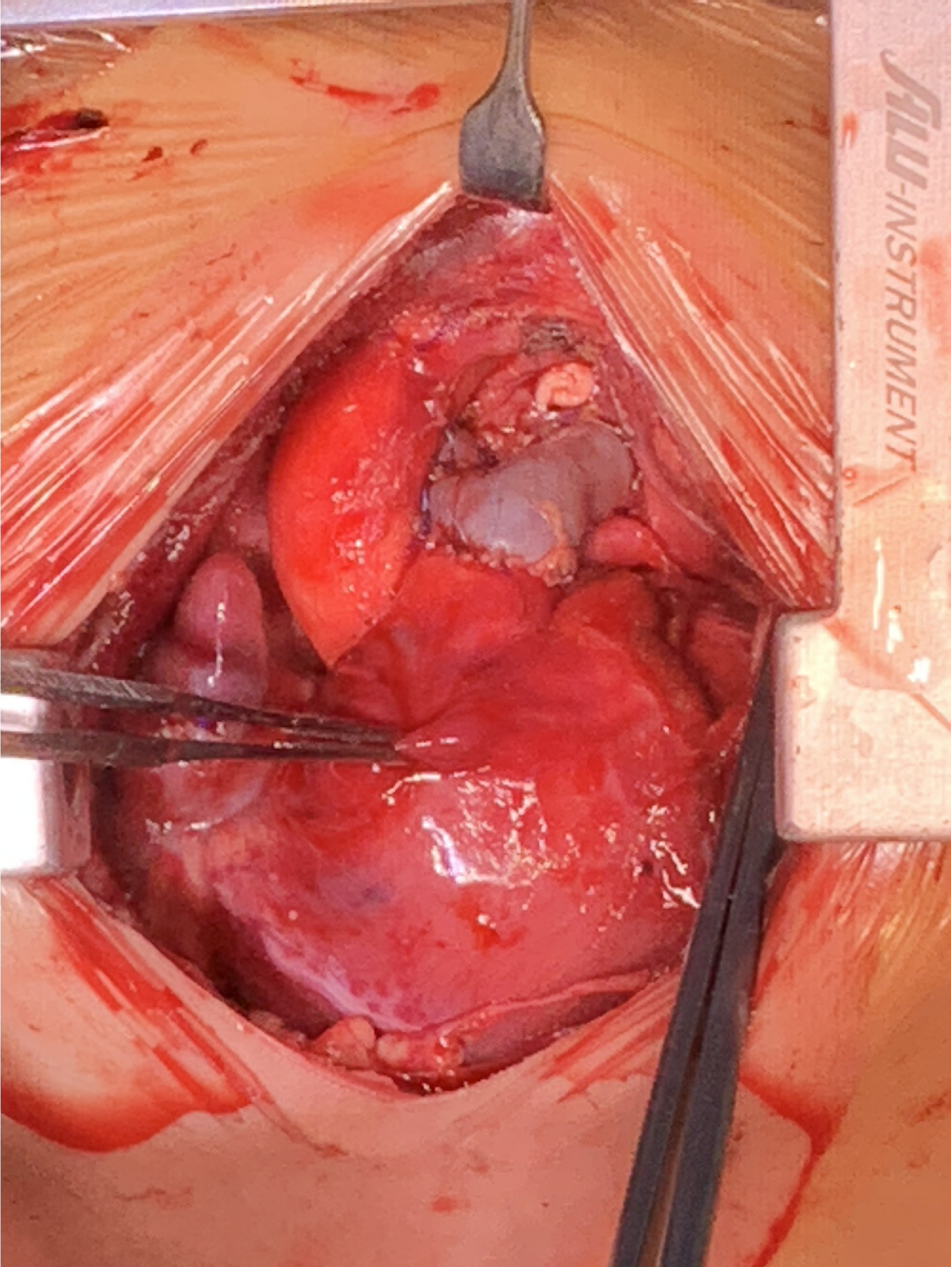
Surgery eventually reconstructed the pulmonary artery with satisfactory morphology and diameters.

After careful evaluation, the diagnosis of UAPA was confirmed, and the operation was electively performed on the patient. After median sternotomy, the pericardium was harvested and treated with glutaraldehyde solution for five minutes. The origin of the right ductus was visualized after the innominate artery mobilization. The distal RPA was located posterior and medial to the SVC. Bilateral branch PAs were completely dissected beyond the lobar division to facilitate a tension-free PA anastomosis. The AAo was also mobilized sufficiently to create adequate space for the neo-RPA. Cardiopulmonary bypass was established *via* AAo and bicaval cannulation. The operation was performed under mild hypothermia (34°C) with a beating heart. After transecting the left ductus, an extension flap was created using the anterior wall of MPA and LPA with the base of the flap located along the right lateral aspect of the MPA and slightly inferior to the level of the proximal LPA (Figure 2A). Care was taken to leave a 5 mm margin of MPA tissue above the sinotubular junction to preserve pulmonary valve function. The distal incision for the extension flap terminated just proximal to the LPA hilum (Figures 2B; 3A). The RPA was then harvested and ductal tissue was completely resected. The RPA was controlled distally with vessel loops on the lobar branches. Exposure was obtained by retracting the vessel loops distally. An incision was made along the inferior aspect of the right lower lobar branch to enlarge the RPA hilum (Figures 2C, 3B). The PA flap was then sutured to the distal segment of the opened RPA to construct the posterior aspect of the neo-RPA using running 7-0 polypropylene sutures (Prolene, Ethicon Inc, USA) (Figure 2C). The inferior, anterior, and superior aspects of the neo-RPA were reconstructed using a glutaraldehyde-treated autologous pericardial patch with a tongue extending into the right lower lobe according to the orientation of the vessel (Figures 2D, 3C). Running 8-0 polypropylene sutures were used for this portion of the PA angioplasty. After completion of the RPA reconstruction, the anterior wall of MPA and LPA was reconstructed with a second treated autologous pericardial patch (Figures 2E, 3D. After finishing the procedure, we successfully reconstructed the PA with satisfactory morphology and diameters (Figure 4). The patient recovered from the surgery uneventfully. Postoperative echocardiography demonstrated no residual vascular shunt and laminar flow in RPA. Postoperative CTA after 1 month revealed normal PA branching patterns and diameter. The patient remains asymptomatic and well upon last follow-up.

It is worth noting that the same surgery was performed on another 3-month-old boy with RPA absence at our institution. The patient also fully recovered from the surgery and showed satisfactory examination results during follow-up.

## Discussion

UAPA is a rare type of congenital abnormality that may coexist with other congenital abnormalities or present as an isolated lesion, the latter form can be asymptomatic (8). RPA absence is more common than LPA absence, and the latter is more frequently associated with fatal cardiovascular abnormalities (4). Those who have only isolated UAPA may present no symptom and can remain asymptomatic into adulthood. It is proposed that UAPA may arise from the persistent connection between the intrapulmonary PA and the distal sixth aortic arch, in addition to the involution of the proximal sixth aortic arch during embryonic development (8).

Diagnosis of UAPA is made upon the basis of a comprehensive medical history, careful physical examination and multi-modality imaging. Most UAPA patients in infancy present with symptoms of pulmonary hypertension or congestive heart failure, while adult patients may mainly complain about exertional dyspnea, recurrent pulmonary infections, haemoptysis and increased exercise intolerance (8). Multi-modality imaging is necessary to establish a UAPA diagnosis, since most imaging features of UAPA are non-specific, whereas angiography remains the gold standard. Although reports on efficacy and benefits of pulmonary vasodilators have been made in UAPA patients, surgery remains the most effective treatment that harbor the potential of curing the abnormality (5).

Previous studies have demonstrated that aggressive early one-stage surgical correction was associated with greater preservation of lung vasculature and improved restoration of pulmonary perfusion (9). El-Hattab et al. have introduced the MPA flap for semi-autologous repair of right-sided UAPA (4); however, this technique has been utilized primarily for left-sided UAPA reconstruction. By comparison, absent RPAs were most commonly reconstructed using a tubular graft, which must be replaced to a size commensurate with the patient's somatic growth (6). Additionally, the proximal incision for the flap should terminate several millimeters above the sinotubular junction to preserve the structural integrity of the pulmonary valve, which may be a particularly important consideration in younger infants. These limitations highlight the inadequacy of using the MPA anterior wall to establish a tension-free reconstruction of the RPA given the latter's discontinuity over a significant distance. Furthermore, in patients with right-sided UAPA, the LPA receives all pulmonary blood flow, which promotes progressive vascular remodeling through elongation and dilatation. Our modified MPA flap angioplasty technique takes advantage of this phenomenon by incorporating the anterior wall of both MPA and LPA into the posterior aspect of the neo-MPA and RPA anastomosis to maximize the extent of autologous tissue used to bridge the gap while simultaneously minimizing anastomotic tension. The anterior wall of the LPA can be easily reconstructed with a separate pericardial patch with excellent geometry after RPA reconstruction.

## Conclusion

Right-sided UAPA with long-gap discontinuity was traditionally considered as one of the most challenging congenital heart malformations for surgeons. We developed a novel technique using MPA and contralateral PA flap combined with fresh autologous pericardium patch for UAPA reconstruction. Short-term postoperative results of two infants with absent RPA were excellent and showed no sign of PA narrowing or pulmonary hypertension. However, growth potential and possibility of stenosis need to be further observed through long-term follow-up.

## Data Availability

The original contributions presented in the study are included in the article/**Supplementary Material**, further inquiries can be directed to the corresponding author/s.
