# Supplementary material for: Case report: Reconstruction of the long-gap unilateral absence of right pulmonary artery with contralateral pulmonary artery flap and autologous pericardial graft
Source: Front Cardiovasc Med. 2023 Mar 8;10:1071111. doi: 10.3389/fcvm.2023.1071111 (PMC10031123; doi:10.3389/fcvm.2023.1071111)
Supplement: Supplementary file 1 [file Table1.docx]

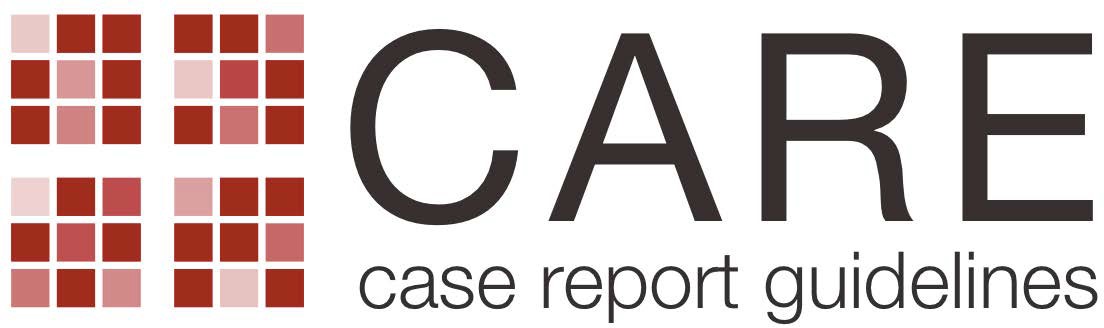
**CARE Checklist of information to include when writing a case report
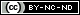
**

**Topic Item Checklist item description Reported on Line**

**Title 1** The diagnosis or intervention of primary focus followed by the words “case report” Page1&2

**Key Words 2** 2 to 5 key words that identify diagnoses or interventions in this case report, including "case report" Page2, sentence23

# Abstract

**(no references)**

**3a** Introduction: What is unique about this case and what does it add to the scientific literature? Page2, sentence15

**3b** Main symptoms and/or important clinical findings Page2, sentence20

**3c** The main diagnoses, therapeutic interventions, and outcomes Page2, sentence21

**3d** Conclusion—What is the main “take-away” lesson(s) from this case? N/A

**Introduction 4** One or two paragraphs summarizing why this case is unique (**may include references**) Page2~3, Introduction

**Patient Information 5a** De-identified patient specific information Page3, sentence13

**5b** Primary concerns and symptoms of the patient Page3, sentence14

**5c** Medical, family, and psycho-social history including relevant genetic information N/A

**5d** Relevant past interventions with outcomes N/A

# Clinical Findings

**Timeline**

**Diagnostic Assessment**

**Therapeutic Intervention**

**Follow-up and Outcomes**

1. Describe significant physical examination (PE) and important clinical findings Page3, sentence14~15
2. Historical and current information from this episode of care organized as a timeline N/A

**8a** Diagnostic testing (such as PE, laboratory testing, imaging, surveys). Page3, sentence 15~23

**8b** Diagnostic challenges (such as access to testing, financial, or cultural) Page3, sentence 24~25

**8c** Diagnosis (including other diagnoses considered) Page3, sentence 24

**8d** Prognosis (such as staging in oncology) where applicable Page4, sentence13~17

**9a** Types of therapeutic intervention (such as pharmacologic, surgical, preventive, self-care) Page3, sentence 25

**9b** Administration of therapeutic intervention (such as dosage, strength, duration) Page4, sentence13~17

**9c** Changes in therapeutic intervention (with rationale) N/A

**10a** Clinician and patient-assessed outcomes (if available) Page4, sentence 14~16

**10b** Important follow-up diagnostic and other test results Page4, sentence17

**10c** Intervention adherence and tolerability (How was this assessed?) N/A

**10d** Adverse and unanticipated events N/A

**Discussion 11a** A scientific discussion of the strengths AND limitations associated with this case report Page4, Discussion

**11b** Discussion of the relevant medical literature **with references** Page4~5, Discussion

**11c** The scientific rationale for any conclusions (including assessment of possible causes) Page5, Discussion

**11d** The primary “take-away” lessons of this case report (without references) in a one paragraph conclusion N/A

**Patient Perspective 12** The patient should share their perspective in one to two paragraphs on the treatment(s) they received N/A

**Informed Consent 13** Did the patient give informed consent? Please provide if requested . . . . . . . . . . . . . . . . . . . . . . . . . . . . . . . . . . . . . . **Yes √ No**
